# Severe community-acquired pneumonia: risk factors for in-hospital mortality

**DOI:** 10.1186/cc10645

**Published:** 2012-03-20

**Authors:** JM Pereira, JA Paiva, JP Baptista, F Froes, J Gonçalves-Pereira

**Affiliations:** 1 Centro Hospitalar S. João, Porto, Portugal; 2 Hospitais Universidade Coimbra, Portugal; 3 Hospital Pulido Valente - CHLN, Lisbon, Portugal; 4 Hospital S. Francisco Xavier, Lisbon, Portugal

## Introduction

Severe community-acquired pneumonia (SCAP) is an important cause of hospital mortality. The goal of this study was to identify variables associated with increased risk of in-hospital mortality at ICU admission.

## Methods

A prospective, multicentre, observational cohort study of all patients with SCAP consecutively admitted to 15 Portuguese ICUs during a 12-month period. Demographic characteristics, co-morbidities, general severity scores (SAPS II, SAPS3, total SOFA), microbiological data and initial empirical antibiotherapy were recorded. Logistic regression analysis was performed to identify predictors of in-hospital mortality.

## Results

A total of 505 (14%) of the 3,572 enrolled patients had SCAP, mostly male (66%) with a median age 58 (29 to 82). Median general severity scores were: SAPS II 44 (21 to 80), SAPS3 65 (41 to 98) and total SOFA 8 (3 to 17). Comorbidities were present in 74% of the patients and the most frequent were: diabetes mellitus (22%), chronic respiratory failure (18%) and alcoholism (15%). Median Charlson's comorbidity index was 4 (0 to 13). At ICU admission, 44% of SCAP patients had septic shock. Thirty-seven per cent of the cases were microbiologically documented (St. pneumoniae - 24%; influenza A (H1N1) virus - 20%; Enterobacteriaceae - 18%) and 12% had secondary bacteremia. Antibiotics were administered in the first 3 hours after hospital admission in 71% of the patients and 76% of them received combination therapy. Antibiotherapy was appropriate in 80% with a median duration of 8 days. Median ICU and hospital lengths of stay were 10 and 19 days respectively. Median ICU and hospital mortalities were 25% and 34% respectively. Variables independently associated with hospital mortality were: SAPS II score (OR 1.06; 95% CI 1.037 to 1.086), severe sepsis (OR 3.61; 95% CI 1.334 to 9.791), septic shock (OR 4.25; 95% CI 1.61 to 11.194), inappropriate antibiotherapy (OR 5.06; 95% CI 1.766 to 14.516) and the use of a macrolide (OR 0.40; 95% CI 0.203 to 0.809).

## Conclusion

Disease severity evaluated by SAPS II and sepsis staging score and inappropriate initial antibiotherapy were independent risk factors for in-hospital mortality. The use of a macrolide was independently associated with a reduced risk of death.

